# Health Care Workers’ Knowledge, Attitudes and Practices on Tobacco Use in Economically Disadvantaged Dominican Republic Communities

**DOI:** 10.3390/ijerph120404060

**Published:** 2015-04-13

**Authors:** Michael G. Prucha, Susan G. Fisher, Scott McIntosh, John C. Grable, Heather Holderness, Kelly Thevenet-Morrison, Zahíra Quiñones de Monegro, José Javier Sánchez, Arisleyda Bautista, Sergio Díaz, Deborah J. Ossip

**Affiliations:** 1 Department of Public Health Sciences, University of Rochester, Medical Center, Rochester, NY 14642, USA; E-Mails: mgprucha@gmail.com (M.G.P.); scott_mcintosh@urmc.rochester.edu (S.M.); heather_holderness@urmc.rochester.edu (H.H.); kelly_thevenet-morrison@urmc.rochester.edu (K.T.-M.); 2 Department of Clinical Sciences, School of Medicine, Temple University, Philadelphia, PA 19140, USA; E-Mail: susan.fisher@tuhs.temple.edu; 3 Department of Medicine, University of Rochester Medical Center, Rochester, NY 14642, USA; E-Mail: john_grable@urmc.rochester.edu; 4 Pontificia Universidad Católica Madre y Maestra, Santiago, Dominican Republic; E-Mails: zquinones@pucmmsti.edu.do (Z.Q.M.); j-javier-s@hotmail.com (J.J.S.); 5 Centro de Atención Primaria Juan XXIII, Santiago, Dominican Republic; E-Mail: arisleydabautista@yahoo.com; 6 Hospital Regional Universitario José María Cabral y Báez, Santiago, Dominican Republic; E-Mail: s_diazpdt@yahoo.com

**Keywords:** tobacco cessation, low-middle income country, primary health care, socioeconomic status, global health

## Abstract

Tobacco use is increasing globally, particularly in low and middle-income countries like the Dominican Republic (DR) where data have been lacking. Health care worker (HCW) interventions improve quit rates; asking patients about tobacco use at each visit is an evidence-based first step. This study provides the first quantitative examination of knowledge, attitudes and practices of DR HCWs regarding tobacco use. All HCWs (*N* = 153) in 7 economically disadvantaged DR communities were targeted with anonymous surveys. Approximately 70% (*N* = 107) completed the primary outcome item, asking about tobacco use at each encounter. Despite >85% strongly agreeing that they should ask about tobacco use at each encounter, only 48.6% reported doing so. While most (94.39%) strongly agreed that smoking is harmful, knowledge of specific health consequences varied from 98.13% for lung cancer to 41.12% for otitis media. Few received training in tobacco intervention (38.32%). Exploratory analyses revealed that always asking even if patients are healthy, strongly agreeing that tobacco causes cardiac disease, and always advising smoke-free homes were associated with always asking. Overall, results demonstrate a disconnect between HCW belief and practice. Though most agreed that always asking about tobacco was important, fewer than half did so. Gaps in HCW knowledge and practices suggest a need for education and policy/infrastructure support. To our knowledge, this is the first reported survey of DR HCWs regarding tobacco, and provides a foundation for future tobacco control in the DR.

## 1. Introduction

As global tobacco use continues to increase, the worst of the associated morbidity and mortality is shifting from high-income countries to low- and middle-income countries (LMICs) [1]. The World Health Organization (WHO) projects eight million annual deaths from tobacco use by 2030, with nearly 80% of these deaths in LMICs [2].

Latin America and the Caribbean (LAC) reflect this trend. The estimated proportionate mortality from tobacco use is 12% globally [3], ranging from 0%–18% among females, and 1%–24% among males in the LAC. The Dominican Republic (DR) shows a mid-range tobacco attributable death rate of 8% among women and 9% among men [1].

DR is a tobacco producing country. It is one of the five countries in the Americas (and one of the three in the LAC) that have not ratified the WHO Framework Convention on Tobacco Control, and the only one that has not even signed the FCTC (as of 25 March 2015) [4]. Of countries with available data, the DR is one of four in the Americas, and of 18 worldwide, dedicating >0.25% of arable land to growing tobacco, and is 11th among all nations dedicating >0.36% of agricultural lands to tobacco (Malawi is first with 2.95%) [1]. Overall tobacco and cigarette use prevalence in the DR ranges from 13.1%–14.9% among adults 18+ (males: 15.6%–17.2%; females: 10.8%–13.0%) [1,2]. We reported tobacco use prevalence at 22.54% (95% CI 20.84–24.24) (males: 27.23%; females: 18.20%) in six economically disadvantaged DR communities (*N* = 2329). Only 5.62% were ex-users [5]. In addition, health care services varied considerably across these communities, thus further reinforcing the importance of preventive care to reduce the need for higher levels of care that may not be available locally.

Research strongly supports the cost-effectiveness of physician interventions for tobacco use. A meta-analysis demonstrated even brief interventions had a relative risk of quitting of 1.66 (95% CI 1.42–1.94) [6] and physician training nearly doubles the odds of patient abstinence through counseling [7]. The 2008 US clinical practice guideline on tobacco use recommended physician counseling and medical treatment of nicotine addiction individually and in combination, with at least minimal interventions (<3 min) for all tobacco users using the 5A model (ask, advise, assess, assist, and arrange), and at least the first 3 A’s with those unwilling to quit. Key to this intervention is asking about tobacco use at all visits [7].

Worldwide, the Global Adult Tobacco Survey (GATS) has demonstrated that tobacco interventions by healthcare providers range from 34.9% to 82.1% across 17 countries based on patient reports [8], while in the US this rate is reported as 48.3% [9]. However, limited data are available from the DR [10]. Within LMICs, training has been targeted largely at physicians and nurses [11], though other health care workers (HCWs) can potentially play a role in tobacco cessation.

Recent data from the Global Health Professions Student Survey (GHPSS) from 47 countries and the Gaza Strip/West Bank show that while over 80% of students surveyed believe that HCWs play a role in advising patients about smoking and should receive training to do so, fewer than 40% had ever received training [12]. Though limited data are available for currently practicing HCWs in the LAC, a survey of Brazilian pulmonologists showed 14% would treat nicotine dependence, and 32.4% would refer their patients to other specialists for such treatment [13]. An older Chilean study found that while 85% of physicians asked their patients about smoking, only 45% did so routinely [14]. In Ecuador, 53.2% of physicians who smoked advised patients to stop smoking, *versus* 71.6% for nonsmoking physicians [15]. In the only two studies from the DR (from our group), qualitative reports by both HCWs and community members indicated no routine HCW assessment of tobacco use, inconsistent knowledge of health effects of tobacco use, limited training in interventions, and no access to smoking cessation medications [10,16]. The lack of access to pharmacotherapy and low prevalence of ex-smokers make these DR communities prime targets for HCW counseling interventions. Our prior research found that HCWs were viewed as key players in tobacco intervention in DR communities [10,16].

The current study is the first to quantitatively assess attitudes, knowledge, training, and practices of HCWs regarding tobacco cessation interventions in the DR, and to examine factors associated with HCWs asking patients about tobacco use.

## 2. Methods

### 2.1. Overview

This study was part of a larger group randomized trial of tobacco control interventions in seven economically disadvantaged DR communities (“Proyecto Doble T2”; PDT2), and was a follow-up to our original PDT1 [5,10]. For the current trial, HCWs in participating communities were surveyed at baseline (April–July 2011) for knowledge, attitudes, and practices regarding tobacco use.

### 2.2. Communities

Seven economically disadvantaged DR communities participated. Three were tobacco growers; four were not. Each housed a Community Technology Center (CTC), directed, at the time, by the Office of the First Lady that served as bases for project operations. The project hired local Site Managers, who were generally also CTC Managers, to coordinate community assessments and interventions under the supervision of DR- and US-based team members.

### 2.3. Healthcare Worker Selection

Site Mangers developed master lists of all HCWs in each community to include general practitioners, *médicos pasantes* (physicians serving one year in communities before beginning specialty training) [10], specialists, dentists, nurses (licensed (RN equivalent) and auxiliary (LPN equivalent)), “nurses by experience”, pharmacists, pharmacy employees, and others. This range reflects the diversity of community HCWs. Health systems varied across communities: three had hospitals, four had health clinics. All HCWs were targeted for surveys.

### 2.4. Survey Development

The survey was based on the original Global Health Professional Survey (GHPS) [17], developed for community clinicians, and later modified for health professions students (GHPSS) because of challenges in surveying community providers. The original GHPS was adapted for cultural applicability for the current study based on our prior PDT1 trial [10,16].

### 2.5. Data Collection

Site Managers were trained in data collection and bioethics by a joint DR-US team. Training was approved by one Institutional Review Board (IRB) in the US and two in the DR. With supervision from DR investigators, Site Managers delivered surveys and a study information sheet in individual envelopes to all HCWs on the master list. Identifiers were removed from the instrument to maintain anonymity, and each provider was assigned a unique identification code to link to subsequent surveys. The questionnaire was self-administered, and placed in a sealed envelope for collection by the site managers about one week later. Site managers re-contacted HCWs who did not return surveys, though HCWs were free to not participate. Consistent with DR IRB regulations, providers were not paid, but were given towels as a gift for returning a completed survey.

### 2.6. Data Management

Completed surveys were kept in the CTCs in locked cabinets until transport to PDT headquarters in Santiago. Data were entered into a password protected, MS Access database, and were checked using a visual verification procedure by the core DR team. Subsequently, the password-protected database was sent to the US team electronically for cleaning. Cleaning involved checking missing data against paper surveys to confirm that responses were not present, and back-coding missing responses where the response was apparent from the skip pattern selected. For example, several participants did not respond to the item, “Have you ever received a formal training in approaches to tobacco cessation to use with your patient/clients?”, but instead followed the skip pattern instructions (“yes (Go to question 49)”) and provided responses to the item (Q49), “What type of tobacco cessation training have you received?”. For these subjects, the response to the initial item was back-coded as “yes”. Reports were returned to the DR team for data entry error corrections. The corrected database was returned to the US for final verifications and analysis.

### 2.7. Measures

Variables were selected for comparison with prior studies, and based on conceptual relationships with HCW intervention. Five categories of variables were included:

*Demographics/Sample Characteristics:* HCW type (physicians, nurses, other), gender, number of years in practice, community;
*Beliefs Regarding Intervention:* Whether HCWs think patients want them to ask, and believe they should routinely ask about tobacco use at adult and pediatric visits and help patients quit, HCW intervention increases quitting and HCWs should set a good example by not smoking;
*Beliefs Regarding Health Effects of Tobacco Use:* Whether HCWs believe tobacco users can improve their health by quitting, smoking and secondhand smoke are harmful to health and a major cause of specific health outcomes (e.g., heart disease, stroke, lung cancer), and thirdhand smoke (toxicants remaining and new toxicants formed when SHS clears) is harmful [18];
*Training/Perceived Skill:* Whether they received tobacco intervention training, perception of their level of knowledge to help patients quit;
*Practice:* How often HCWs ask patients if they use tobacco, advise patients to quit, advise patients to quit if you think their illness is tobacco related, advise patients to quit if you think their illness is not tobacco related, advise patients to quit if they are healthy, advise females to quit if they are pregnant, assist patients to quit, and advise patients to have smoke-free homes and vehicles.

### 2.8. Data Analysis

Inclusion of surveys for analysis was contingent upon completion of the primary outcome item: How often HCWs ask about tobacco use. Data were analyzed using SAS version 9.3 for Windows.First, descriptive data were compiled to examine knowledge, attitudes, beliefs and practices of HCWs regarding tobacco. Second, bivariate analyses were conducted to examine relationships between individual variables and whether HCWs report “always” asking about tobacco use, *versus* asking with any other level of frequency (4-point Likert scale). This was chosen as the primary outcome based on the best practice guidelines for HCWs, asking about tobacco use at *every* patient encounter [7]. Pearson chi-square analyses were used unless the expected cell size was <5, in which case a Fisher’s exact test was used. Scaled variables were dichotomized as “Strongly Agree” or “Always,” *versus* all other responses (5 or 4-point Likert scale respectively). Though some studies have combined categories (e.g., “Strongly Agree”/”Somewhat Agree” or “Always”/”Often”) [19,20,21], others have used only the end point (e.g., “Strongly Agree” or “Every Visit”) in comparison to all else [21,22,23]. The current study used the latter more conservative dichotomy to at least partly account for HCW over-reporting, based on prior literature [20] and on our own prior data that had indicated low levels of knowledge and practice by HCWs [10,16]. Number of years in practice was categorized as <1 year, 1–10 years, 11–20 years, 21–30 years and 30+ years, and a Mantel-Haenszel chi-square test was used. Specific health outcomes beliefs were examined first as individual, dichotomous variables with chi-square or Fisher’s exact tests, then as a continuous variable, tabulating the total number of “Strongly Agree” responses by provider, using a *t*-test to examine the relationship. With the available sample size for the key outcome (Ask always; *N* = 107), chi-square analyses would detect a 30% difference in proportions with 0.80 power and α = 0.05.

Finally, variables with *p* < 0.10 in the bivariates were included in stepwise logistic regressions to build the most parsimonious model for factors related to HCWs “always” asking about tobacco use. Initial multivariable models controlled for number of years in practice, gender, and community or tobacco-growing community (the latter two variables controlled for clustering of HCWs within communities). Neither community variable was significant in any run and they were eliminated from subsequent runs. A final set of models was run including additional advising practices for tobacco use.

## 3. Results

Of the 153 HCWs targeted, 109 (71.2%) returned surveys and 107 (69.93%) completed the primary outcome item (asking about tobacco use). The number of HCWs/community ranged from 6–24. HCW characteristics are presented in Table 1.

**Table 1:** Subject Characteristics (*N* = 107).

Characteristic	N	% Overall
Gender		
Female	85	80.19
Male	21	19.81
Time in Practice		
≤6 years	26	26.26
7–12 years	25	25.25
13–20 years	24	24.24
≥21 years	24	24.24
Institution Type **^a^**		
Public	92	85.98
Private/Non-Governmental	24	22.43
Other	1	0.93
HCW Type		
*Nurses*	*51*	*49.04*
Auxiliary	38	36.54
Licensed	12	11.54
By Experience	1	0.96
*Physicians*	*30*	*28.85*
General Practice	12	11.54
Medico Pasante **^b^**	5	4.81
Pediatrician	3	2.88
OB/GYN	5	4.81
Cardiologist	1	0.96
Internal Medicine	1	0.96
Surgery	1	0.96
Specialist(unknown)	1	0.96
Dentist	1	0.96
*Other* **^c^**	*23*	*22.12*
Tobacco Use Status		
Current User	4	3.74
Former User	6	5.61
Never User	82	76.64
No Response	15	14.02
Reported Patient Tobacco Use **^d^**		
Cigarettes	85	79.44
Cigars	55	51.40
Self-Rolled	40	37.38
Smokeless	43	40.19
Pipe	30	28.04
Received formal training on tobacco interventions	41	38.32
Report very sufficient knowledge on tobacco use and intervention strategies	40	37.38

**^a^** Some HCWs hold multiple jobs therefore category >100% (*n* > 107); **^b^** First year doctors who are assigned to areas in need by the government of the Dominican Republic; **^c^** Includes community public health promoters, public health supervisors, and pharmacy workers; **^d^** Providers may report patients use more than one type of tobacco therefore category >100%.

Respondents were mostly female (80.19%), which is consistent with national data on healthcare workers in the DR [24]. Respondents worked largely at public institutions (85.98%) for <1 to >21 years, were largely never smokers (76.64%), and saw patients who used a range of tobacco products. Few had received formal tobacco treatment training (38.32%), and only 37.38% felt their knowledge about tobacco use and cessation was very sufficient.

Table 2 and Table 3 represent HCW knowledge of health conditions related to tobacco, attitudes about tobacco interventions with patients, and practices in tobacco interventions. While nearly all (94.39%) strongly agreed that smoking was harmful to health, knowledge of specific health conditions varied. Over 90% strongly agreed that smoking is a major cause of lung cancer, emphysema, and laryngeal cancer and 80% strongly agreed that smoking increases TB mortality. However, relative to knowledge levels for these conditions, fewer strongly agreed that smoking during pregnancy increases risk of miscarriage (79.41%), and that smoking is a major cause of cardiac disease (76.70%) and stroke (68.63%), and fewer than half recognized smoking as a major cause of bladder cancer (43.56%).

HCWs demonstrated somewhat less knowledge of secondhand smoke (SHS) effects. About three-quarters strongly agreed that SHS increases risk of respiratory tract illnesses in children (78.85%), and lung cancer (75.00%) and heart disease in nonsmokers (70.00%); relatively fewer strongly agreed that SHS increases risk of sudden infant death syndrome (SIDS; 62.38%) and otitis media in children (41.18%). Most (83.02%) thought that smokeless tobacco was harmful to health, and about three quarters believed that thirdhand smoke was harmful to infants and children. Most 85.98% strongly agreed that tobacco users could improve their health if they quit. Only about half (53.85%) strongly agreed that a patient’s chance of quitting increases if they are advised by an HCW, and only one-third (33.33%) strongly agreed that patients want their advice.

**Table 2:** Health care workers’ knowledge of the health effects of smoking, secondhand smoke, and smokeless tobacco.

Item	Strongly Agree % (n)	Somewhat Agree % (n)	Somewhat Disagree % (n)	Strongly Disagree % (n)	Not Sure % (n)
Smoking…					
*Is a major cause of lung cancer*	98.13 (105)	0.93 (1)	-	0.93 (1)	-
*Is a major cause of emphysema*	95.19 (99)	2.88 (3)	-	1.92 (2)	-
*Is harmful to health*	94.39 (101)	2.80 (3)	-	2.80 (3)	-
*Is a major cause of laryngeal cancer*	93.40 (99)	5.66 (6)	-	0.94 (1)	-
*Increases risk of death from TB*	80.00 (84)	13.33 (14)	1.90 (2)	2.86 (3)	1.90 (2)
*During pregnancy increases miscarriage risk*	79.41 (81)	16.67 (17)	1.96 (2)	0.98 (1)	0.98 (1)
*Is a major cause of cardiac disease*	76.70 (79)	18.45 (19)	1.94 (2)	1.94 (2)	0.97 (1)
*Is a major cause of stroke*	68.63 (70)	23.53 (24)	0.98 (1)	-	6.86 (7)
*Is a major cause of bladder cancer*	43.56 (44)	33.66 (34)	8.91 (9)	5.94 (6)	7.92 (8)
Secondhand Smoke Increases Risk of…					
*Respiratory tract illness in children*	78.85 (82)	19.23 (20)	-	1.92 (2)	-
*Lung cancer in nonsmokers*	75.00 (78)	16.35 (17)	2.88 (3)	0.96 (1)	4.81 (5)
*Heart disease in nonsmokers*	70.00 (70)	22.00 (22)	1.00 (1)	1.00 (1)	6.00 (6)
*Sudden Infant Death Syndrome*	62.38 (63)	23.76 (24)	3.96 (4)	2.97 (3)	6.93 (7)
*Otitis media in children*	41.18 (42)	31.37 (32)	7.84 (8)	6.86 (7)	12.75 (13)
Breathing air in a room today where someone smoked yesterday is harmful to infants/children	75.96 (79)	16.35 (17)	2.88 (3)	2.88 (3)	1.92 (2)
Smokeless tobacco is harmful to health	83.02 (88)	15.09 (16)	0.94 (1)	0.94 (1)	-
Tobacco users can improve their health if they quit	85.98 (92)	7.48 (8)	-	2.80 (3)	3.74 (4)

For subsequent analyses “strongly agree” and “always” were compared with all others combined.

**Table 3:** Health care workers’ attitudes and behaviors on tobacco use advising practices.

Item	Strongly Agree % (n)	Somewhat Agree % (n)	Somewhat Disagree % (n)	Strongly Disagree % (n)	Not Sure % (n)
A patient’s chance of quitting increases if a HCW advises them to quit	53.85 (56)	34.62 (36)	1.92 (2)	2.88 (3)	6.73 (7)
Patients want you to advise them to stop using tobacco	33.33 (35)	41.90 (44)	14.29 (15)	3.81 (4)	6.67 (7)
HCWs like you should…					
* Set a good example by not using tobacco*	92.31 (96)	4.81 (5)	-	1.92 (2)	0.96 (1)
* Routinely ask patients about tobacco use*	89.22 (91)	7.84 (8)	1.96 (2)	-	0.98 (1)
* Routinely ask parents about tobacco use during pediatric visits*	86.41 (89)	13.59 (14)	-	-	-
* Routinely help patients to quit using tobacco*	83.81 (88)	15.24 (16)	0.95 (1)	-	-
*Routinely advise patients who use tobacco to quit*	80.58 (83)	11.65 (12)	-	6.80 (7)	0.97 (1)
How often…	**Always**	**Often**	**Rarely**	**Never**	
* Do you ask about tobacco use?*	49.53 (53)	35.51 (38)	7.48 (8)	7.48 (8)	
* Do you advise patients to quit using tobacco?*	64.49 (69)	22.43 (24)	2.80 (3)	10.28 (11)	
* Do you advise female patients to quit using tobacco if they are pregnant?*	75.70 (81)	13.08 (14)	1.87 (2)	9.35 (10)	
* Do you advise patients if you think an illness is related to tobacco use?*	70.09 (75)	16.82 (18)	2.80 (3)	10.28 (11)	
* Do you advise patients to have smoke-free homes?*	60.75 (65)	23.36 (25)	6.54 (7)	9.35 (10)	
* Do you advise patients to have smoke-free vehicles?*	59.81 (64)	22.43 (24)	8.41 (9)	9.35 (10)	
* Do you advise patients to quit using tobacco if they are healthy?*	54.21 (58)	28.04 (30)	5.61 (6)	12.15 (13)	
* Do you assist tobacco users to quit?*	54.29 (57)	31.43 (33)	4.76 (5)	9.52 (10)	
* Do you advise patients if you do NOT think an illness is related to tobacco use?*	52.38 (55)	23.81 (25)	10.48 (11)	13.33 (14)	

For subsequent analyses, “strongly agree” and “always” were compared with all others combined.

Additionally, while 89.22% of HCWs strongly agreed that they should routinely ask about tobacco use, only 49.53% reported always doing so. Similarly, although 80.58% strongly agreed that they should routinely advise patients who use tobacco to quit, only 64.49% reported always doing so. Variability was found in conditions under which HCWs reported always advising patients to quit. About three-quarters reported always advising if the patient is pregnant or they think an illness is related to tobacco use, but only about half always advised if they do not think the illness is related to tobacco use or if the patient is healthy. Fewer than two-thirds reported always advising patients to have smoke-free homes (60.75%) and vehicles (59.81%).

Variables significantly (*p* < 0.05) associated with always asking about tobacco use in bivariate analyses (Table 4) were strongly agreeing that smoking is a major cause of bladder cancer, and that SHS increases the risk of SIDS, along with a number of variables related to advising practices.

HCWs who always asked about tobacco use also strongly agreed with a higher mean number of health conditions (mean 6.53, SD 1.59; possible range 0–8) associated with tobacco use compared with those who did not always ask (mean 5.83, SD 1.87; *p* = 0.041). Agreeing that smoking is a major cause of cardiac disease was of borderline significance (*p* = 0.07). No differences in asking were found for any other health beliefs, provider demographics, or provider training.

Logistic regression results varied depending on whether other advising practices regarding patient tobacco use were included in the model. The Hosmer-Lemeshow chi-square was not significant for any, indicating an adequate fit for the models. When not including other tobacco use advising practices, the only factor associated with always asking about tobacco use was always advising about smoke-free homes (OR 4.15, 95% CI 1.61–10.69). When including other tobacco use advising practices, the only factors associated with always asking were belief that tobacco use is a major cause of cardiac disease (OR 4.18, 95% CI 1.14–15.35), and always advising patients to quit if they are healthy (OR 12.32, 95% CI 4.20–36.18).

**Table 4:** Bivariate analysis of factors associated with asking about tobacco use **^1^**.

Variable	Total ^2^	Ask about Tobacco Use at Every Encounter n (%)	Ask with Any Other Level of Frequency n (%)	*p* Value
Total (*n* = 107)		53 (49.53)	54 (50.47)	
Gender				0.223
Male	21 (19.81)	8 (38.10)	13 (61.90)	
Female	85 (80.19)	45 (52.94)	40 (47.06)	
Community				0.280 **^3^**
1	20 (18.69)	10 (50.00)	10 (50.00)	
2	14 (13.08)	5 (35.71)	9 (64.29)	
3	21 (19.63)	8 (38.10)	13 (61.90)	
4	10 (9.35)	3 (30.00)	7 (70.00)	
5	12 (11.21)	7 (58.33)	5 (41.67)	
6	6 (5.61)	4 (66.67)	2 (33.33)	
7	24 (22.43)	16 (66.67)	8 (33.33)	
Tobacco Producing				0.931
Yes	48 (44.86)	24 (50.00)	24 (50.00)	
No	59 (55.14)	29 (49.15)	30 (50.85)	
Provider Type				0.301
MD	30 (28.85)	16 (53.33)	14 (46.67)	
Nurse	51 (49.04)	27 (52.94)	24 (47.06)	
Other	23 (22.12)	8 (34.78)	15 (65.22)	
Years in Practice				0.513
≤6 Years	26 (24.30)	11 (42.31)	15 (57.69)	
7–12 Years	25 (23.36)	10 (40.00)	15 (60.00)	
13–20 Years	24 (22.43)	15 (62.50)	9 (37.50)	
≥21 Years	24 (22.43)	13 (54.17)	11 (45.83)	
No Response	8 (7.48)	4 (50.00)	4 (50.00)	
Smoking is a major cause of cardiac disease				0.0702
Strongly Agree	79 (76.70)	43 (54.43)	36 (45.57)	
Any Other	24 (23.30)	8 (33.33)	16 (66.67)	
Smoking is a major cause of bladder cancer				0.0147
Strongly Agree	44 (43.56)	28 (63.64)	16 (36.36)	
Any Other	57 (56.44)	22 (38.60)	35 (61.40)	
SHS increases risk of SIDS				0.0374
Strongly Agree	63 (62.38)	35 (55.56)	28 (44.44)	
Any Other	38 (37.62)	13 (34.21)	25 (65.79)	
Advise patients to quit tobacco				<0.0001
Always	69 (64.49)	46 (66.67)	23 (33.33)	
Any Other	38 (35.51)	7 (18.42)	31 (81.58)	
Advise patients to quit if illness thought to be tobacco related				0.0002
Always	75 (70.09)	46 (61.33)	29 (38.67)	
Any Other	32 (29.91)	7 (21.88)	25 (78.13)	
Advise patients to quit if illness NOT thought to be tobacco related				0.0003
Always	55 (52.38)	37 (67.27)	18 (32.73)	
Any Other	50 (47.62)	16 (32.00)	34 (68.00)	
Advise patients to quit tobacco if they are healthy				<0.0001
Always	58 (54.21)	41 (70.69)	17 (29.31)	
Any Other	49 (45.79)	12 (24.49)	37 (70.69)	
Advise females to quit tobacco if they are pregnant				0.0004
Always	81 (75.70)	48 (59.26)	33 (40.74)	
Any Other	26 (24.30)	5 (19.23)	21 (80.77)	
Assist patients to quit using tobacco				<0.0001
Always	57 (54.29)	41 (71.93)	16 (28.07)	
Any Other	48 (45.71)	11 (22.92)	37 (77.08)	
Advise patients to have smoke-free homes				0.0005
Always	65 (60.75)	41 (63.08)	24 (36.92)	
Any Other	42 (39.25)	12 (28.57)	30 (71.43)	
Advise patients to have smoke-free vehicles				0.0011
Always	64 (59.81)	40 (62.50)	24 (37.50)	
Any Other	43 (40.19)	13 (30.23)	30 (69.77)	

Results shown only for *a priori* covariates (gender, community, tobacco producing community, provider type, and years in practice) and variables significant at *p* < 0.10; **^1^** Analyses completed using Chi-square unless noted; **^2^** Totals may vary depending on response rate for individual variables; **^3^** Fisher’s Exact Test.

## 4. Discussion

To our knowledge, this is the first quantitative study of HCW knowledge, attitudes, beliefs and practices in the Dominican Republic, and the results are concerning. Notably, fewer than half of all respondents ask about tobacco use at every encounter (49.53%), and just under two-thirds (64.49%) report always advising patients to quit, despite overwhelming evidence demonstrating the efficacy of these practices. The rate of always asking is slightly higher than rates from a 16 country study, in which 41% of physicians overall discussed smoking at all visits [21], comparable to rates of always asking reported by HCWs in Turkey (48.9%) [22], but lower than documented rates of screening in the United States (63.6%) [25]. The rate of always advising is lower than the 84.6%–89.9% of physicians in the 16 country study who reported always advising [21], and also lower than always advising rates in Turkey (83.6%) [22] and Italy (81.1%) [23].

Additionally, gaps were identified in HCWs’ knowledge about the specific health effects of tobacco use and SHS exposure on their patients. Although over 90% strongly agreed that smoking is a major cause of lung and laryngeal cancer, as well as emphysema, relatively fewer strongly agreed that smoking is a major cause of cardiac disease (76.70%), and fewer than half strongly agreed that SHS increases risk of otitis media. The higher recognition of lung cancer risks is consistent with data from England and Germany, with variable findings for these two countries in knowledge regarding chronic obstructive lung disease [26]. Similarly, HCWs in Turkey reported high knowledge of the effects of smoking on lung cancer and chronic cough, but little knowledge of other smoking-caused diseases [22].

Reported interventions in the current study are at suboptimal levels; for example, although three-quarters reported always advising pregnant smokers to quit, this should be near or at 100% for this vulnerable population, and only about half advised patients to quit if they thought the illness was unrelated to tobacco use. As in other countries [17], few had received formal training in interventions or felt they had sufficient knowledge regarding tobacco use and interventions. In contrast to prior research showing a relationship between provider training and increased intervention [27,28,29], formal training was not related to always asking about tobacco use in the current study. It is not clear what type of education was received by the low percentage of providers who reported receiving training. Although information on risks of tobacco may be incorporated in some health sciences coursework in the DR, there did not appear to be any formal curricula or professional workshops on tobacco interventions outside of the current project. These gaps are typical for much of the LAC region [30]. Providing such formal and continuing education to HCWs in specific risks of tobacco use and evidence-based interventions may provide a first step toward consistent intervention. As part of the subsequent intervention component of the current trial, investigators provided community-based HCW training in tobacco cessation interventions, as well as regional and national level training to HCWs through public health and professional society partnerships.

HCWs demonstrated a disconnect between beliefs and practices, with most believing they should ask and intervene but fewer than half always asking and about two-thirds always advising. The higher percentage of those reporting advising relative to asking may have been due to providers not asking patients who had indicated tobacco use (or no tobacco use) previously, or to earlier qualitative reports by HCWs that they “could tell” if someone smoked and therefore did not need to ask [10]. The latter strategies may have resulted in missing patients who used tobacco. Building systems to support intervention, such as having a formal tobacco screening protocol in place, and using this as a prompt to intervene with tobacco users [7], along with support from professional societies and credentialing agencies, may increase HCW practices regarding tobacco use. Finally, only about half strongly agreed that HCW advice increased patients’ chances of quitting, and only one third strongly agreed that patients wanted them to provide such advice. Prior research, including qualitative research by our team, has demonstrated that patients do want HCW advice to quit [16,31]. Providing feedback to HCWs on the acceptability of such advice may increase HCWs willingness to intervene.

Three variables were associated with always asking about tobacco use in the final multivariate models: Always advising about smoke-free homes, always asking even if patients are healthy and belief that tobacco use was a major cause of cardiac disease. Notably, type of HCW and other provider characteristics were not related to always asking. The relationship with advising smoke-free homes may reflect a clustering of intervention practices. In addition, it is possible that indicating always asking even when patients are healthy reflects a greater understanding of the long-term risks of tobacco use, which may have influenced HCWs to engage in more consistent asking about tobacco use.

There are limitations to this study. The sample size was relatively small (though reflective of all HCWs in these communities), thus limiting power for comparisons. In addition, only seven communities were surveyed, thus the generalizability to the broader group of HCWs in the DR is unknown. Notably, however, within these 7 communities, the response rate was high (71%), perhaps due to features of the survey methodology (hand-delivered and collected surveys, use of sealed envelopes and no identifiers for anonymity, follow-up with non-completers), project team engagement with HCWs for the project, and/or use of local data collectors. Finally, data were self-reported, which may have resulted in over-reporting of knowledge and/or intervention practices based on social desirability [20]. Indeed, the rates of reported knowledge and practices were markedly higher than those identified in our earlier qualitative research [10,16] and survey data based on patient report (data to be reported separately). For this reason, we selected only “Strongly Agree” and “Always” and compared these with all other answers. It is possible that this use of a single endpoint underestimated provider knowledge and practice relative to trials that combined, for example, “Strongly” and “Somewhat” for the “Agreed” category. Indeed, the literature includes studies that vary in their use of one or the other categorization. Although our choice limits comparisons with studies that used the combined category [19,20,21], it is consistent with our prior data [10,16] and allows direct comparison with studies that used a comparable single endpoint [21,22,23]. Finally, larger studies of HCWs in the DR are needed to expand the evidence base for policy and educational recommendations to sustain and improve the work that is already being accomplished by those who have taken up this important public health issue.

## 5. Conclusions

This study is unique in that it provides the first quantitative report of HCW knowledge, beliefs, and practices regarding tobacco use in the DR, and reflects community-based HCWs in economically disadvantaged communities. Results indicate gaps in knowledge and practices of HCWs, indicating a need to increase provider knowledge about health risks of tobacco use and intervention efficacy, and to further assess and provide feedback to HCWs on the acceptability of their interventions to patients. Increased training in evidence based interventions for all providers, both in school and on the job, and a supporting infrastructure to initiate and sustain HCW practice change to consistently ask about tobacco use and intervene at every patient encounter are essential components for overall DR tobacco control.

## Supplementary Files

Supplementary File 1
