# Supplementary material for: Health Care Workers’ Knowledge, Attitudes and Practices on Tobacco Use in Economically Disadvantaged Dominican Republic Communities
Source: Int J Environ Res Public Health. 2015 Apr 13;12(4):4060–75. doi: 10.3390/ijerph120404060 (PMC4410233; doi:10.3390/ijerph120404060)
Supplement: Supplementary File 1 [file ijerph-12-04060-s001.pdf]

Health Care Workers' Knowledge, Attitudes and Practices on Tobacco Use in Economically Disadvantaged Dominican Republic Communities

---

**Sondeo de Proveedores de Salud**<br>**Proyecto Doble T**<br>Proyecto Control de Tabaquismo en Colaboración con la Comunidad	Fecha __ __ / __ __ / __ __ __ __<br>Código de la comunidad __ __<br>Código del Proveedor __ __ __ __ __ __<br>Código del Administrador del Sondeo __ __ __ __ __ __

**Información Demográfica del Proveedor de Salud**			
**P1**	¿Qué tipo de proveedor de salud es usted? **[Por favor marque una opción]**<br>□ Médico Pasante<br>□ Médico General<br>□ Pediatra<br>□ Gineco-Obstetra<br>□ Especialista, especifique ______________<br>□ Dentista<br>□ Licenciada en Enfermería<br>□ Auxiliar de Enfermería<br>□ Enfermera por "Experiencia"<br>□ Boticario<br>□ Empleado de Farmacia<br>□ Otro, por favor especifique ____________________	**P2**	¿Dónde practica? **[Por favor marque todas las que apliquen]**<br>□ Institución pública<br>□ Institución privada<br>□ Organización no-gubernamental<br>□ Otro, por favor especifique ___________________
		**P3**	¿Cuánto tiempo ha estado practicando como proveedor de salud?<br>___ Número de años **O** ___ Número de meses
		**P4**	¿Cuál es su género?<br>□ Masculino □ Femenino

**Conocimientos y Actitudes** [Por favor encierre en un círculo la opción que mejor corresponda]						
	**Fumar…**	**Fuertemente de acuerdo**	**Algo de acuerdo**	**Algo en desacuerdo**	**Fuertemente en desacuerdo**	**No estoy segur@**
**P5**	…es dañino para la salud.	1	2	3	4	6
**P6**	…es una causa mayor de enfermedad cardíaca.	1	2	3	4	6
**P7**	…es una causa mayor de accidente cerebrovascular.	1	2	3	4	6
**P8**	…es una causa mayor de cáncer de pulmón.	1	2	3	4	6
**P9**	…es una causa mayor de cáncer de laringe (garganta).	1	2	3	4	6
**P10**	…es una causa mayor de cáncer de vejiga.	1	2	3	4	6
**P11**	…es una causa mayor de enfisema pulmonar.	1	2	3	4	6
**P12**	…incrementa el riesgo de morir por tuberculosis.	1	2	3	4	6
**P13**	…durante el embarazo aumenta el riesgo de aborto.	1	2	3	4	6
	**El humo de segunda mano (respirar el humo de otra persona) aumenta el riesgo…**	**Fuertemente de acuerdo**	**Algo de acuerdo**	**Algo en desacuerdo**	**Fuertemente en desacuerdo**	**No estoy segur@**
**P14**	… de enfermedad cardíaca en no fumadores.	1	2	3	4	6
**P15**	… de cáncer de pulmón en no fumadores.	1	2	3	4	6
**P16**	… del Síndrome de Muerte Súbita Infantil.	1	2	3	4	6
**P17**	… de enfermedades del tracto respiratorio en niños.	1	2	3	4	6
**P18**	… de otitis media (infecciones del oído medio) en niños.	1	2	3	4	6
**P19**	Respirar el aire en una habitación **hoy** donde alguien fumó **ayer** es dañino para la salud de los niños e infantes.	1	2	3	4	6
**P20**	El tabaco sin humo (el que se usa olido, masticado o puesto en la boca) es dañino para la salud.	1	2	3	4	6
**P21**	Los usuarios de tabaco pueden mejorar su salud si dejan de usarlo.	1	2	3	4	6

Intervenciones de los Proveedores de Salud [Por favor encierre en un círculo la opción que mejor corresponda]						
		Fuertemente de acuerdo	Algo de acuerdo	Algo en desacuerdo	Fuertemente en desacuerdo	No estoy segur@
**P22**	La probabilidad de dejar de usar tabaco de sus pacientes / clientes se incrementa si un proveedor de salud como usted le aconseja dejarlo.	**1**	**2**	**3**	**4**	**6**
**P23**	Los pacientes / clientes quieren que **usted** les aconseje que dejen de usar tabaco.	**1**	**2**	**3**	**4**	**6**
	**Los proveedores de salud como usted deben rutinariamente…**					
**P24**	… preguntar sobre los hábitos de uso de tabaco de sus pacientes / clientes.	**1**	**2**	**3**	**4**	**6**
**P25**	…aconsejar a sus pacientes / clientes que usan tabaco para dejarlo.	**1**	**2**	**3**	**4**	**6**
**P26**	… ayudar a sus pacientes a dejar de usar tabaco.	**1**	**2**	**3**	**4**	**6**
**P27**	… aconsejar a sus pacientes / clientes que tengan hogares libres de humo.	**1**	**2**	**3**	**4**	**6**
**P28**	… aconsejar a sus pacientes / clientes que tengan vehículos libres de humo.	**1**	**2**	**3**	**4**	**6**
**P29**	…preguntar durante visitas pediátricas si los padres fuman.	**1**	**2**	**3**	**4**	**6**
**P30**	… poner un buen ejemplo no usando productos de tabaco.	**1**	**2**	**3**	**4**	**6**

Políticas (Regulaciones) [Por favor encierre en un círculo la opción que mejor corresponda]						
		Fuertemente de acuerdo	Algo de acuerdo	Algo en desacuerdo	Fuertemente en desacuerdo	No estoy segur@
**P31**	Fumar no debe permitirse en los espacios cerrados.	**1**	**2**	**3**	**4**	**6**
**P32**	Fumar no debe permitirse en los centros de salud.	**1**	**2**	**3**	**4**	**6**
**P33**	Debe haber etiquetas de advertencias más fuertes en los paquetes de cigarrillos.	**1**	**2**	**3**	**4**	**6**
**P34**	La venta de productos de tabaco a menores debe estar prohibida.	**1**	**2**	**3**	**4**	**6**

Prácticas de Consejería
[Por favor encierre en un círculo la opción que mejor corresponda]

	¿Con qué frecuencia…	Nunca	Rara vez	Con frecuencia	Siempre
**P35**	…le pregunta a sus pacientes / clientes si ellos usan tabaco?	1	2	3	4
**P36**	… le aconseja a sus pacientes / clientes que dejen de usar tabaco?	1	2	3	4
**P37**	… le aconseja a sus pacientes / clientes que dejen de usar tabaco si piensa que su enfermedad está relacionada al uso de tabaco?	1	2	3	4
**P38**	…le aconseja a sus pacientes / clientes que dejen de usar tabaco si tienen una enfermedad que usted **no** cree que está relacionada al uso de tabaco?	1	2	3	4
**P39**	…le aconseja a sus pacientes / clientes que dejen de usar tabaco si ellos están saludables?	1	2	3	4
**P40**	… le aconseja a sus pacientes / clientes femeninas que dejen de usar tabaco si ellas están embarazadas?	1	2	3	4
**P41**	…usted asiste a sus pacientes / clientes en dejar de usar tabaco?	1	2	3	4
**P42**	...le aconseja a sus pacientes / clientes que tengan casas libre de humo?	1	2	3	4
**P43**	…le aconseja a sus pacientes / clientes que tienen vehículos que los mantengan libre de humo?	1	2	3	4

P44 ¿Tiene conocimiento sobre recursos / intervenciones para dejar de fumar/usar tabaco en esta comunidad?

□ Sí **[Vaya a la pregunta P45]** □ No **[Vaya a P46]**

P45 ¿Qué tipo de recursos están disponibles para dejar de fumar o usar tabaco en esta comunidad?

- □ Especialistas en cesación de uso de tabaco
- □ Consejería
- □ Ayuda en el Internet
- □ Charlas
- □ Medicaciones para dejar de fumar
- □ Materiales de autoayuda (brochures o afiches)
- □ Remedios caseros
- □ Clases para dejar de fumar
- □ Ferias de salud Antitabaquismo
- □ Videos o presentaciones sobre el uso de tabaco y su cesación

P46 ¿Ha dado alguna charla en esta comunidad sobre el uso de tabaco o cesación en los últimos 3 meses?

□ Sí □ No

P47 ¿Cree usted que el conocimiento que tiene sobre el uso de tabaco / cesación es suficiente para que usted pueda aconsejar a sus pacientes a dejar de usar tabaco?

- □ Muy suficiente
- □ Algo suficiente
- □ Algo insuficiente
- □ Muy insuficiente

**P48**	¿Alguna vez ha recibido un entrenamiento formal en el manejo de la cesación de uso de tabaco para usarlo con sus pacientes / clientes?<br>□ Sí **[Vaya a la pregunta P49]** □ No **[Vaya a la pregunta P50]**
**P49**	¿Qué tipo de entrenamiento en cesación de uso de tabaco ha recibido?<br>□ Entrenamiento formal durante su carrera universitaria<br>□ Entrenamiento formal durante sus estudios de postgrado<br>□ Otro, por favor especifique ___________________<br>□ Entrenamiento específico, simposios, conferencias o talleres en esta comunidad<br>□ Entrenamiento específico, simposios, conferencias o talleres fuera de esta comunidad
**P50**	Si se pusieran a su disposición sesiones de entrenamiento gratuitas sobre el tópico de ayudar a sus pacientes a dejar de fumar, ¿estaría interesado en participar?<br>□ Sí □ No

Descriptores de sus Pacientes / Clientes

**P51**	Principalmente, ¿qué tipo de pacientes ve usted en su práctica diaria? **[Por favor marque todas las opciones que apliquen.]**<br>□ Niños (0-12)<br>□ Adolescentes (13-17)<br>□ Adultos jóvenes (18-44)<br>□ Adultos de edad mediana (45-64)<br>□ Adultos mayores / envejecientes (>65)<br>□ Embarazadas	**P52**	En general, ¿qué tipo(s) de tabaco usan sus pacientes? **[Por favor marque todas las opciones que apliquen]**<br>□ Cigarrillos<br>□ Enrollados por sí mismos (túbano, pachuché, cigarrito, pirulí, hueva)<br>□ Pipa<br>□ Cigarros<br>□ Masticado / Tabaco sin humo<br>□ Hookah (Pipa de agua)<br>□ Otro, por favor especifique ___________________

Su Exposición al Tabaco

[Por favor encierre en un círculo la opción que corresponda en cada caso.]

P53 ¿Cuál de las afirmaciones en el encabezado describe mejor las reglas sobre el uso de tabaco en los siguientes lugares?

		**Fumar nunca es permitido**	**Fumar es permitido en cualquier lugar**	**Fumar es permitido solo a algunas personas**	**Fumar es permitido solo en algunos lugares**	**No hay reglas**
P53a	Su casa	**1**	**2**	**3**	**4**	**5**
P53b	Su principal lugar de trabajo	**1**	**2**	**3**	**4**	**5**
P53c	Su principal carro / camioneta / jeeppeta	**1**	**2**	**3**	**4**	**5**

P54 ¿Cómo describiría su uso de tabaco?

□ Nunca he usado tabaco **[Vaya a la pregunta P62, "Pregunta para todos" al final del sondeo.]**

□ Actualmente uso tabaco **[Vaya a la pregunta P55, "Preguntas para Fumadores / Usuarios de tabaco"]**

□ Dejé de usar tabaco **[Vaya a la pregunta P59, "Preguntas para Ex – fumadores / Ex - usuarios de tabaco"]**

Preguntas para Fumadores / Usuarios de Tabaco

P55 ¿A qué edad empezó a usar productos de tabaco? __ __

P56 ¿Con qué frecuencia usa usted de cada uno de los productos que se enlistan a continuación?
[Por favor marque las casillas que correspondan a su uso. Si NO usa ese producto, marque No lo uso.]

Tipo de Tabaco	**Uso diario**	**No Uso diario**	**No lo uso**
Cigarrillos	□	□	□
Enrollados por sí mismo (túbano, pachuché, cigarrito, pirulí, hueva, otro)	□	□	□
Pipa	□	□	□
Cigarros	□	□	□
Masticado / Tabaco sin humo	□	□	□
Hookah (Pipa de agua)	□	□	□
Otro, especifique ________________	□	□	□

P57 ¿Cuál de las siguientes le describe mejor?
[Por favor marque una opción.]

□ No estoy list@ para dejarlo en los próximos 6 meses.

□ Estoy pensando en dejarlo en los próximos 6 meses.

□ Estoy list@ para dejarlo ahora

P58 ¿Ha intentado dejar de fumar / usar tabaco?

□ Sí **[vaya a P61]** □ No **[vaya a P62]**

Preguntas para ExFumadores/ExUsuarios de Tabaco

P59 ¿A qué edad empezó a usar productos de tabaco? __ __

P60 ¿Con qué frecuencia usaba usted de cada uno de los productos que se enlistan a continuación?
[Por favor marque las casillas que correspondan a su uso. Si NO usa ese producto, ponga No lo usaba.]

Tipo de Tabaco	**Usaba diario**	**No Usaba diario**	**No lo usaba**
Cigarrillos	□	□	□
Enrollados por sí mismo (túbano, pachuché, cigarrito, pirulí, hueva, otro)	□	□	□
Pipa	□	□	□
Cigarros	□	□	□
Masticado / Tabaco sin humo	□	□	□
Hookah (Pipa de agua)	□	□	□
Otro, especifique ________________	□	□	□

[Vaya a P61]

**P61**	¿Cómo lo dejó? **[Por favor marque todas las opciones que apliquen.]**	
	□ Dulces, mentas	□ Asistencia de un proveedor de salud
	□ Voluntad propia	□ Materiales de autoayuda (brochures, afiches)
	□ TRN (terapia de reemplazo de nicotina)	□ Sitio de Internet
	□ Champix	□ Especialista en Cesación de Uso de Tabaco
	□ Otros medicamentos, especifique _______________	□ Charlas
	□ Remedios caseros	□ Clases de Cómo Dejar de Fumar
	□ Reducción diaria (disminuir el número de cigarrillos)	□ Ferias de Salud Anti-Tabaquismo
	□ Religión	□ Videos o presentaciones acerca del tabaquismo/cesación
	□ Familia	□ Otro, especifique ______________________________
	□ Amigos	

**Pregunta para Todos**	
**P62**	¿Hay algo más que quisiera decirnos?

Usted ha completado exitosamente el Sondeo de Proveedores de Salud del Proyecto Doble T 2. Su tiempo e información son extremadamente valiosos y se mantendrán como confidenciales. GRACIAS nueva vez por su tiempo y participación en nuestro proyecto. Favor de poner el sondeo en el sobre y sellar el sobre.

© 2015 by the authors; licensee MDPI, Basel, Switzerland. This article is an open access article distributed under the terms and conditions of the Creative Commons Attribution license (http://creativecommons.org/licenses/by/4.0/).
